# Fibroblast-Derived Decellularized Extracellular Matrix as a Bioactive Substrate for Osteoblast Activation

**DOI:** 10.1021/acsomega.5c13590

**Published:** 2026-06-05

**Authors:** Devy F Garna, Agata Szubska, Sama Salman, Aisha Mohammed, Lucy Di-Silvio

**Affiliations:** King’s College London-Guy’s Campus. Centre for Oral, Clinical and Translational Sciences, 4616 Guy’s Hospital Tower Wing, Floor 17th, Great Maze Pond, London SE1 1UL, United Kingdom

## Abstract

The extracellular matrix (ECM) plays a critical role in regulating cell behavior and is increasingly incorporated into biomaterial design. However, the reproducible generation of cell-derived ECM remains challenging due to variability in decellularization methods. This study refines a detergent-based protocol for preparing fibroblast-derived ECM and evaluates its effects on primary human osteoblasts (HOBs). Modifications to detergent exposure and processing conditions enhanced DNA removal (up to 98.3%) while preserving protein content, with residual DNA predominantly below 100 bp. Proteomic analysis was included as a descriptive, contextual reference and is not directly linked to functional results. Functional assays demonstrated that fibroblast-derived ECM modulated osteoblast responses in a concentration-dependent manner. A concentration of 1.25 mg/mL increased alkaline phosphatase activity and significantly increased RUNX2 levels, indicating enhanced early osteogenic signaling. Mineralization assessment at day 14 using Alizarin Red S and von Kossa staining revealed increased calcium and phosphate deposition in dECM-treated cultures compared with controls, suggesting progression toward matrix mineralization. Overall, the fibroblast-derived dECM provides a biologically active substrate that supports osteoblast function and osteogenic responses. Further studies are required to fully establish its osteogenic potential.

## Graphical abstract

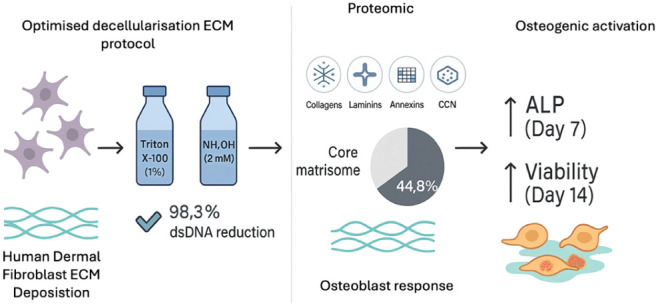

## Introduction

The extracellular matrix (ECM) provides essential biochemical and structural cues that regulate cell adhesion, proliferation, differentiation, and tissue homeostasis.^1–5^ In addition to its mechanical role, the ECM acts as a dynamic signaling network that influences lineage specification and tissue repair. Cell-derived ECM (cECM) has consequently attracted increasing attention as a biomaterial platform due to its capacity to recapitulate features of native cellular microenvironments.^1–5^

A major challenge in producing cECM is the decellularization process, which aims to remove cellular and nuclear material while preserving the matrix itself. Detergent-based methods, including Triton X-100, NH_4_OH, and NaOH, are commonly used because they effectively lyse cells while retaining many ECM proteins.^1–3^ Enzymatic treatments, such as DNase or RNase, are often added to improve nuclear clearance.^1,2,4^ Despite their widespread use, these protocols vary considerably in detergent concentration, exposure duration, culture conditions, and washing procedures, resulting in inconsistent ECM yield and quality. Reported exposure times range from seconds to hours, frequently without systematic comparison or quantitative assessment of nuclear removal.^2,4^ However, these protocols differ widely in reagent concentrations, exposure times, and washing steps, leading to variability in ECM quality. In some cases, residual detergent may remain and affect cell behavior, whereas excessive washing can damage the matrix structure, which is generally more fragile than tissue-derived ECM.^1,6^ This variability complicates reproducibility and limits the ability to consistently meet established decellularization criteria of ≤50 ng dsDNA mg^–1^ dry weight and <200 bp fragment length.^7,8^

The utility of the cell-derived ECM lies in its adaptability and compatibility with modern biomaterial fabrication strategies. Decellularized matrices can be lyophilized and incorporated into hydrogels, bioinks, or porous scaffolds, providing versatile platforms for regenerative applications.^9^ These systems have demonstrated osteoinductive and angiogenic potential, particularly when derived from osteogenic or stem cell lineages.^10^ Nonetheless, the extent to which decellularization parameters influence ECM bioactivity and protein composition remains insufficiently defined.

For bone-related applications, the ECM composition is a key determinant of biological function. Fibroblast-derived ECM is enriched in fibrillar collagens (types I and III), fibronectin, vitronectin, and hyaluronic acid, contributing to structural integrity and wound healing processes.^1,11^ In contrast, osteoblast-derived ECM contains mineral-associated proteins such as osteocalcin and osteonectin, supporting matrix mineralization.^10^ While fibroblast-derived ECM provides a practical and reproducible platform for biomaterial development, it lacks intrinsic osteogenic specificity compared to osteoblast-derived ECM. Osteoblast-derived ECM is enriched in mineralization-associated proteins and serves as a biologically relevant reference for the composition of the osteogenic matrix.^2,3^ In this context, proteomic profiling of the osteoblast-derived ECM can provide a reference framework for identifying matrix-associated proteins linked to osteogenic function. Importantly, the present study does not directly compare fibroblast- and osteoblast-derived ECM. Instead, osteoblast-derived ECM is used to contextualize proteomic findings and to support the interpretation of matrix-associated components relevant to osteogenic activity. This approach enables functional evaluation of the fibroblast-derived ECM while maintaining a biologically informed reference for osteogenic relevance.

To better interpret ECM-associated biological function, characterization of matrix composition is required. Advances in high-resolution proteomics, including LC–MS/MS, now enable the comprehensive identification of core matrisome and associated proteins.^12,13^ However, a key gap remains in linking decellularization conditions with proteomic alterations and functional cellular responses. In particular, the relationship between detergent-based processing of fibroblast-derived ECM and osteoblast behavior remains poorly understood. In this context, proteomic profiling of osteoblast-derived ECM serves as a reference for identifying matrix-associated proteins relevant to osteogenic function.

While this study focuses on early osteogenic responses, primarily assessed through alkaline phosphatase activity and biochemical characterization, mineralization assays were also performed to further evaluate osteogenic potential. To address this, the present study applies a controlled detergent-based approach to prepare fibroblast-derived ECM with reduced nuclear content while preserving key matrix components. Through integrated biochemical characterization and functional assays in primary human osteoblasts (HOBs), we evaluate how fibroblast-derived ECM influences cell viability and alkaline phosphatase activity, an early osteogenic indicator. This work provides a methodological framework for generating biologically active cECM substrates and contributes to the development of ECM-based strategies for bone tissue engineering.

## Results

An overview of the workflow is presented in Figure 1, outlining ECM production, decellularization, biochemical characterization, proteomic analysis, and osteoblast functional assessment.

**Figure 1:**
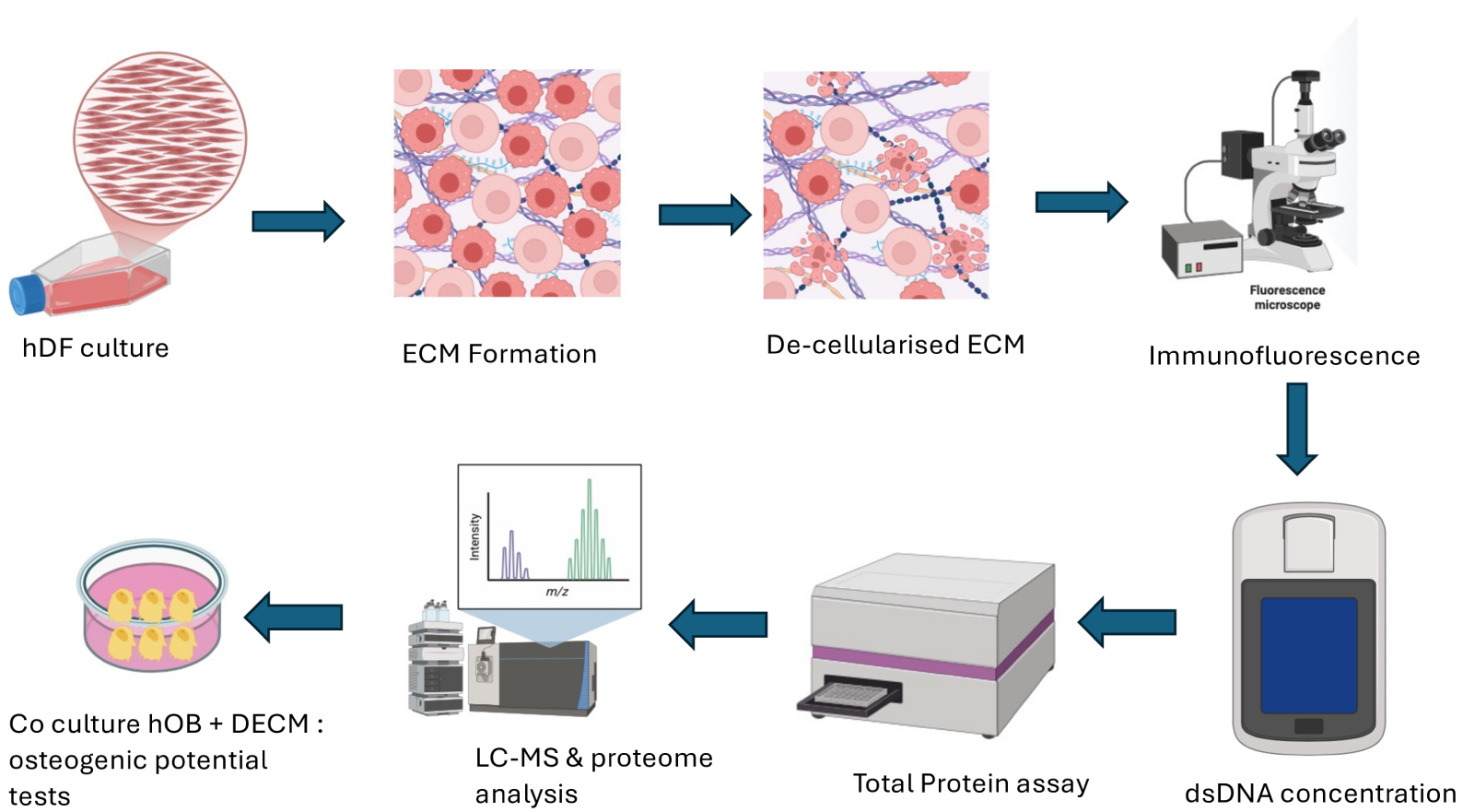
Schematic overview of the experimental workflow. Human dermal fibroblasts (hDFs) were cultured to produce extracellular matrix (ECM), which was subsequently decellularized to obtain cell-free dECM. The decellularized matrix was characterized by immunofluorescence imaging, total protein quantification, and dsDNA assays. Proteomic profiling was performed using LC–MS/MS, and the biological function of the dECM was evaluated by coculturing with primary human osteoblasts (HOBs) to assess osteogenic potential. This figure was created using BioRender.com.

### Evaluation of Preliminary Decellularization Conditions

Initial decellularization using Triton X-100 and NH_4_OH resulted in incomplete nuclear removal and disruption of fibronectin architecture (Figure 2A–D). Residual Hoechst-positive nuclei were consistently observed, and DNA quantification exceeded accepted decellularization thresholds (>50 ng mg^–1^), indicating insufficient removal of nuclear material.

**Figure 2:**
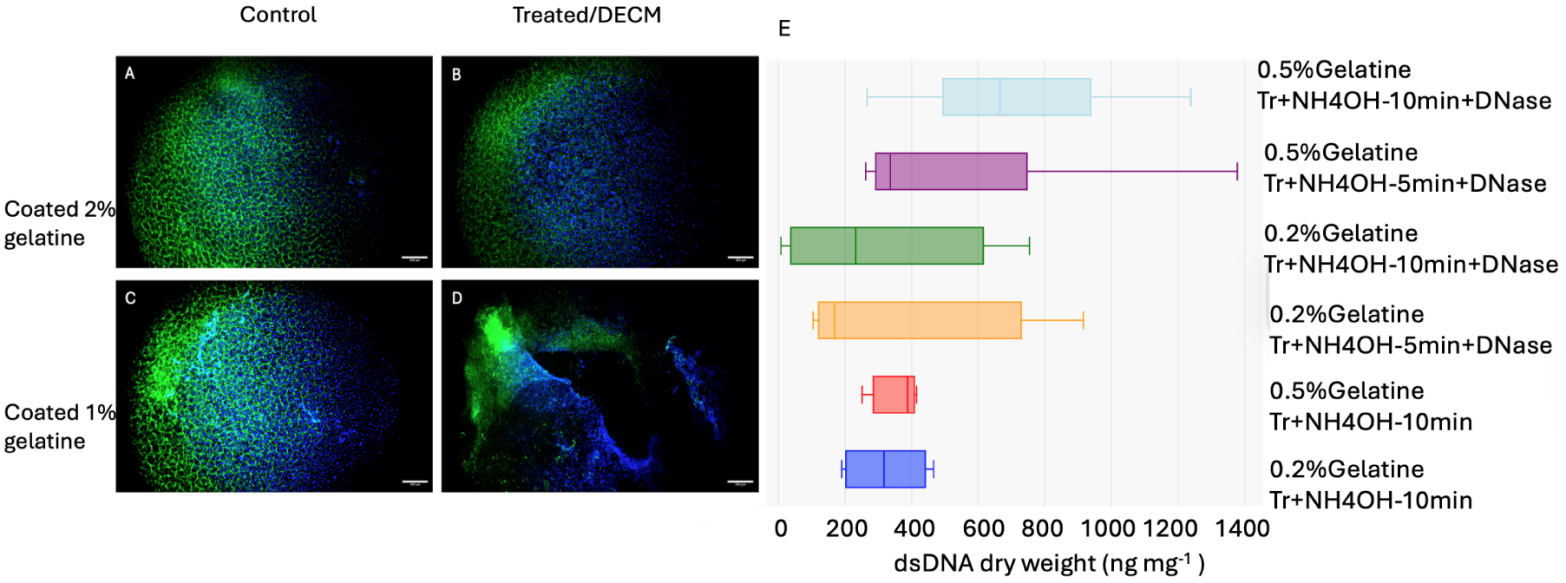
Evaluation of preliminary decellularization protocol on fibroblast-derived extracellular matrix (ECM). (A–D) Representative immunofluorescence images of human dermal fibroblast (HDF)-derived ECM stained for fibronectin (green) and nuclei (Hoechst, blue). Cells were cultured for 4 days to generate early-stage ECM and subjected to decellularization using 0.5% Triton X-100 and 1.4 mM NH_4_OH (equivalent to 1/14 of 20 mM) for 10 min at 37 °C. Panels show: (A) control, 2% gelatin; (B) decellularized, 2% gelatin; (C) control, 1% gelatin; and (D) decellularized, 1% gelatin. Residual Hoechst-positive nuclei are visible in treated samples, indicating incomplete removal of nuclear material, while disruption of fibronectin fibers suggests partial loss of ECM structure. Scale bar = 300 μm. (E) Quantification of dsDNA content measured by NanoDrop for samples subjected to different reagent combinations (Tr, Triton X-100; NH_4_OH, ammonium hydroxide; DNase I, 10 μL mL^–1^ for 30 min). All conditions exceeded the accepted decellularization threshold (≤50 ng DNA mg^–1^ dry weight), confirming insufficient nuclear removal under these preliminary conditions. Data are presented as box-and-whisker plots (*n* = 5).

It should be noted that these preliminary experiments were conducted at an earlier time point in culture (day 4), when ECM deposition was less mature. This difference in matrix maturity likely contributed to variability in both structural integrity and decellularization efficiency, thereby limiting direct comparison with later conditions.

### Refinement of Decellularization Conditions

Subsequent experiments incorporated adjustments in culture duration (days 5–8), detergent exposure, and substrate stabilization. These modifications improved ECM retention and reduced DNA content by up to 98.3% relative to control (Figure 3B). Protein content was better preserved in samples cultured on stabilized substrates (Figure 3A), suggesting that substrate fixation contributed to maintaining ECM integrity during detergent treatment. No statistically significant differences were observed between the fixed and nonfixed groups under combined detergent conditions, indicating that detergent composition, rather than substrate conditions, was the dominant factor influencing DNA removal. To further assess decellularization quality, DNA fragmentation was analyzed by agarose gel electrophoresis (Figure 3C). Residual DNA fragments were predominantly below 100 bp, consistent with established decellularization criteria (<200 bp).

**Figure 3:**
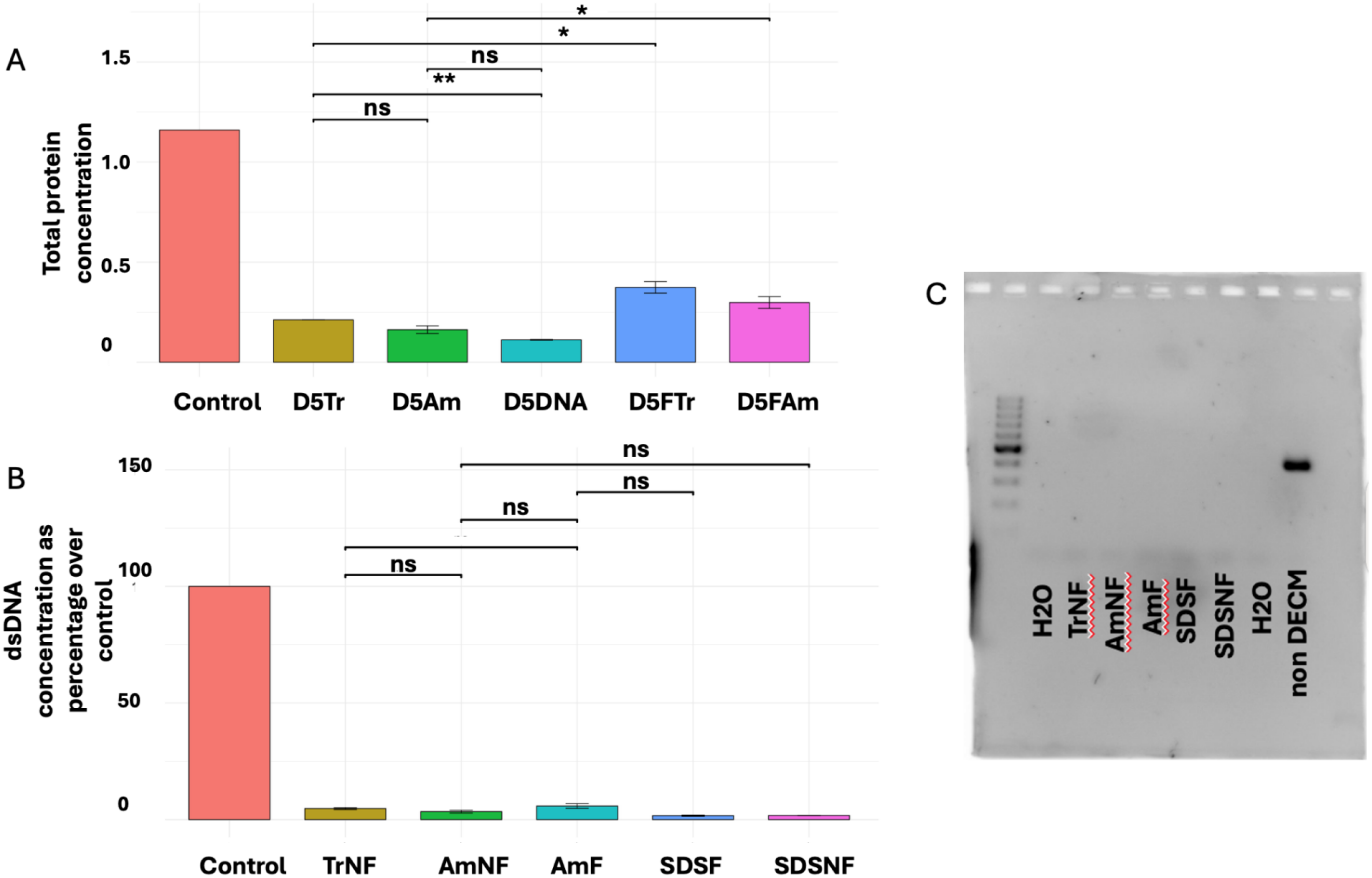
Biochemical characterization of fibroblast-derived ECM following refinement of decellularization conditions. (A) Total protein content of ECM following different detergent treatments. (B) Residual double-stranded DNA (dsDNA) content following decellularization under varying conditions. Data are presented as mean ± SD (*n* = 4). Culture time points reflect differences in ECM maturity and are not directly comparable. (C) Agarose gel electrophoresis showing DNA fragment size following decellularization. DNA fragments are predominantly below 100 bp, indicating effective DNA degradation. Abbreviations: Tr, Triton X-100; Am, ammonium hydroxide (NH_4_OH); SDS, sodium dodecyl sulfate; D5, day 5 culture; F, fixed substrate; NF, nonfixed substrate; DNA, DNase-treated condition.

### Proteomic Characterization of the ECM

Proteomic analysis identified 49 ECM-associated proteins, including collagens, laminins, annexins, and CCN family members (Figures 4–5). Histone and cytoskeletal proteins were also detected, suggesting the presence of residual intracellular components despite substantial DNA reduction.

**Figure 4:**
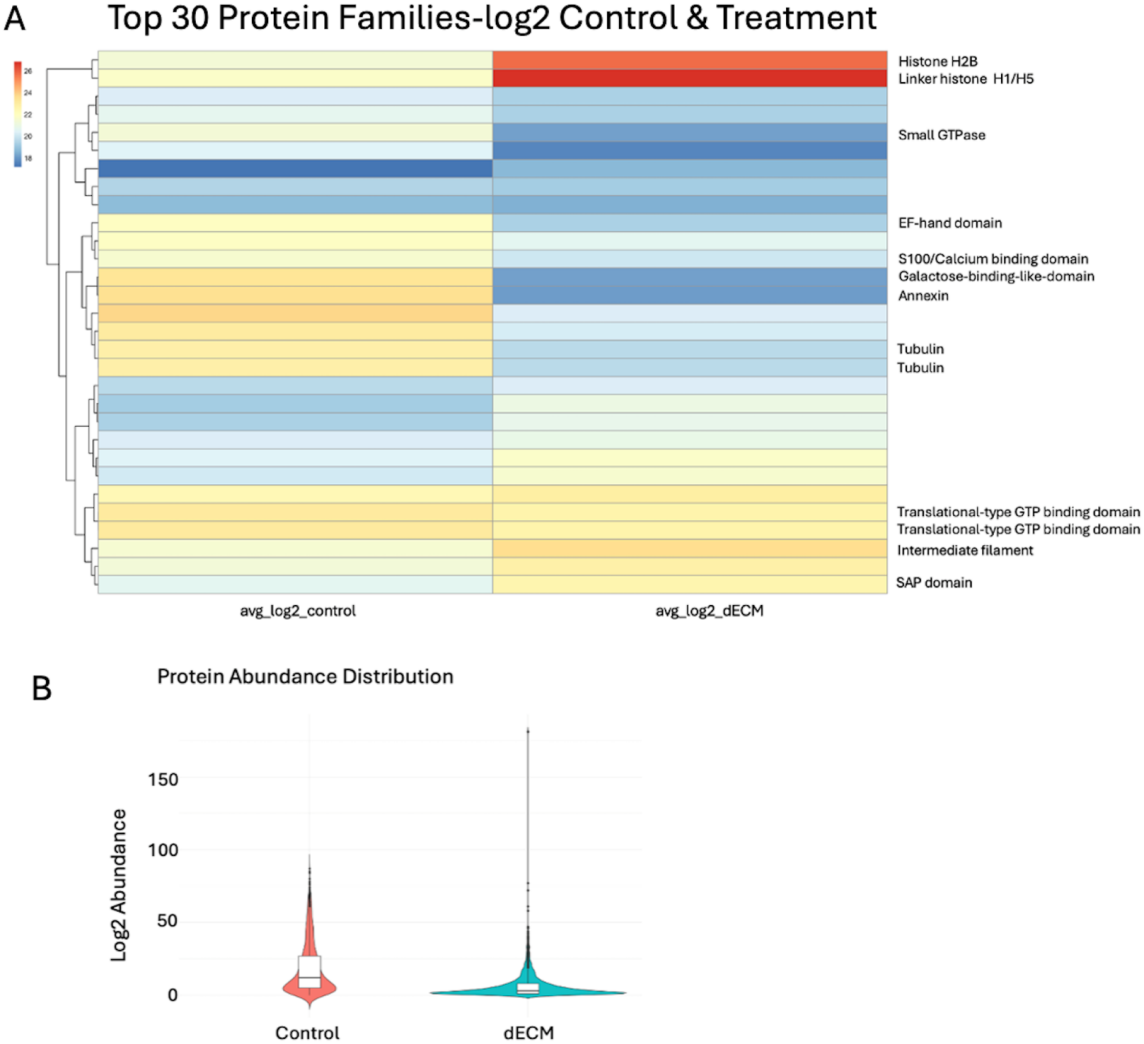
Comparative proteomic profiling of human osteoblast-derived ECM (HOBS dECM) and untreated control ECM. (A) Heatmap showing the top 30 most abundant protein families identified in control and decellularized ECM (dECM) samples based on log_2_-transformed label-free quantification (LFQ) intensity values. Proteins were filtered at a false discovery rate (FDR) of *q* ≤ 0.01 and required ≥2 unique peptides and ≥2 peptide-spectrum matches (PSMs). Higher relative abundance is indicated by red coloration, and lower abundance by blue. (B) Violin plot illustrating overall protein abundance distribution between control and dECM groups. The broader distribution observed in the dECM group reflects increased proteomic diversity following decellularization.

**Figure 5:**
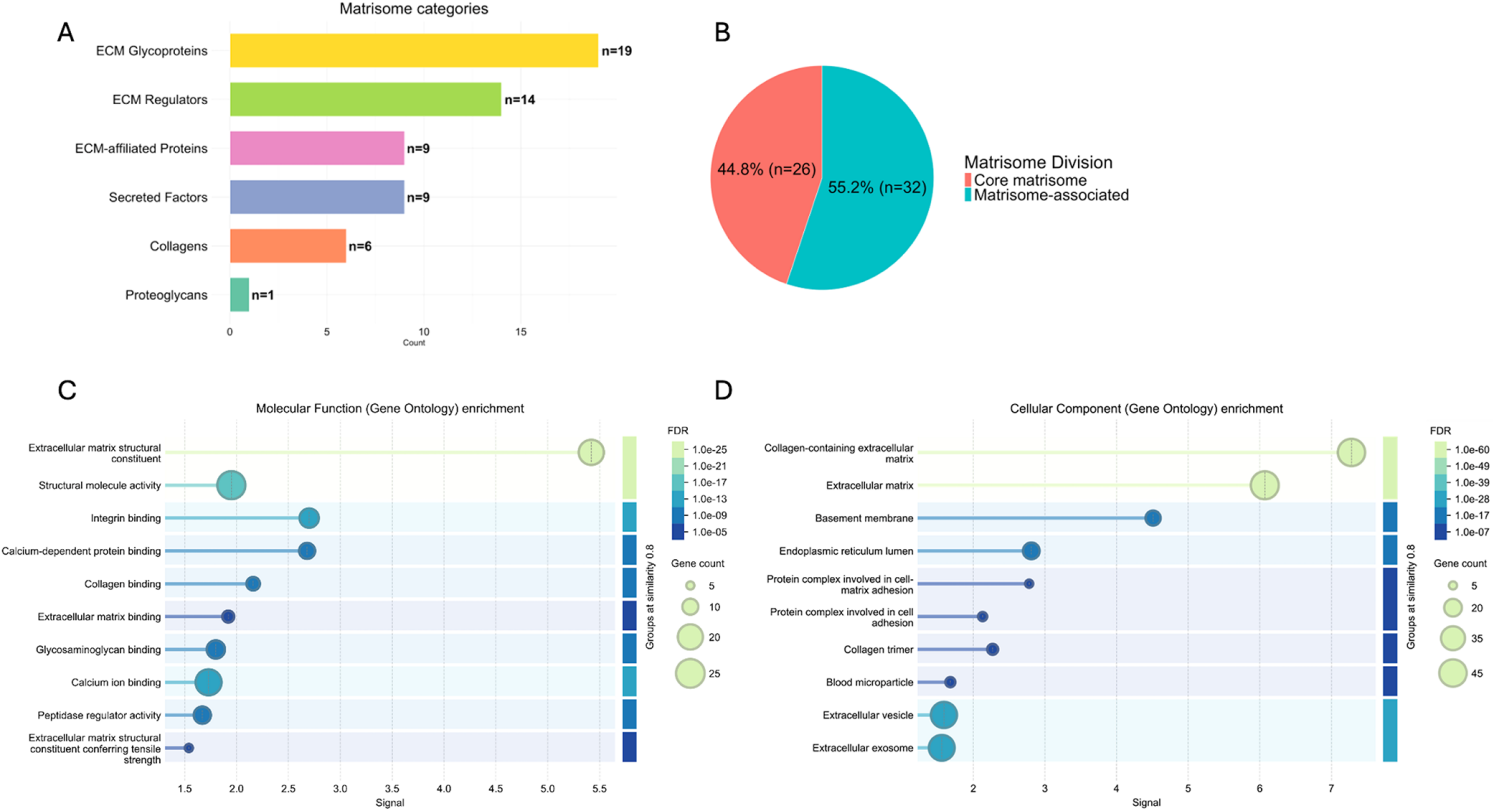
Matrisome composition and gene ontology (GO) enrichment analysis of decellularized human osteoblast-derived ECM (HOBS dECM). (A) Classification of ECM-associated proteins identified by LC–MS/MS into matrisome categories, including ECM glycoproteins, ECM regulators, ECM-affiliated proteins, collagens, proteoglycans, and secreted factors. (B) Distribution of identified ECM-associated proteins into core matrisome (44.8%) and matrisome-associated (55.2%) groups, based on Matrisome Project annotation. Percentages represent the proportion of proteins within the ECM-associated data set. (C) Gene ontology (GO) enrichment analysis of molecular function terms and (D) cellular component terms. Bubble size indicates the number of proteins assigned to each category, and color intensity represents statistical significance (false discovery rate, FDR-adjusted *p*-values ranging from 1.0 × 10^–25^ to 1.0 × 10^–6^). Enrichment values are based on precursor ion intensity. Proteomic analysis was performed on osteoblast-derived ECM to provide contextual information on matrix-associated proteins relevant to osteogenic function and is presented as a descriptive analysis.

Matrisome classification indicated that 44.8% of identified proteins belonged to the core matrisome, with the remainder classified as matrisome-associated components. Gene ontology analysis showed enrichment in ECM structural organization, integrin binding, and calcium ion binding. Because biological replicates were pooled prior to analysis, these findings are descriptive and should be interpreted as qualitative insights rather than statistically validated differences. Several protein families, including histones, intermediate filaments, and annexins, were identified with variable abundance patterns across samples; however, because samples were pooled prior to analysis, these observations remain descriptive.

### Cell Viability of HOBs Cultured with dECM

Cell viability of primary human osteoblasts (HOBs) cultured with different concentrations of decellularized extracellular matrix (dECM) was assessed using the Alamar Blue assay at days 1, 7, and 14 (Figure 6). A two-way mixed ANOVA revealed a significant main effect of dECM concentration (*F*(3,36) = 8.35, *p* < 0.001, partial η^2^ = 0.41), a significant main effect of time (*F*(2,72) = 61.42, *p* < 0.001), and a significant interaction between time and concentration (*F*(6,72) = 25.12, *p* < 0.01), indicating that the effect of dECM concentration on metabolic activity varied over time.

**Figure 6:**
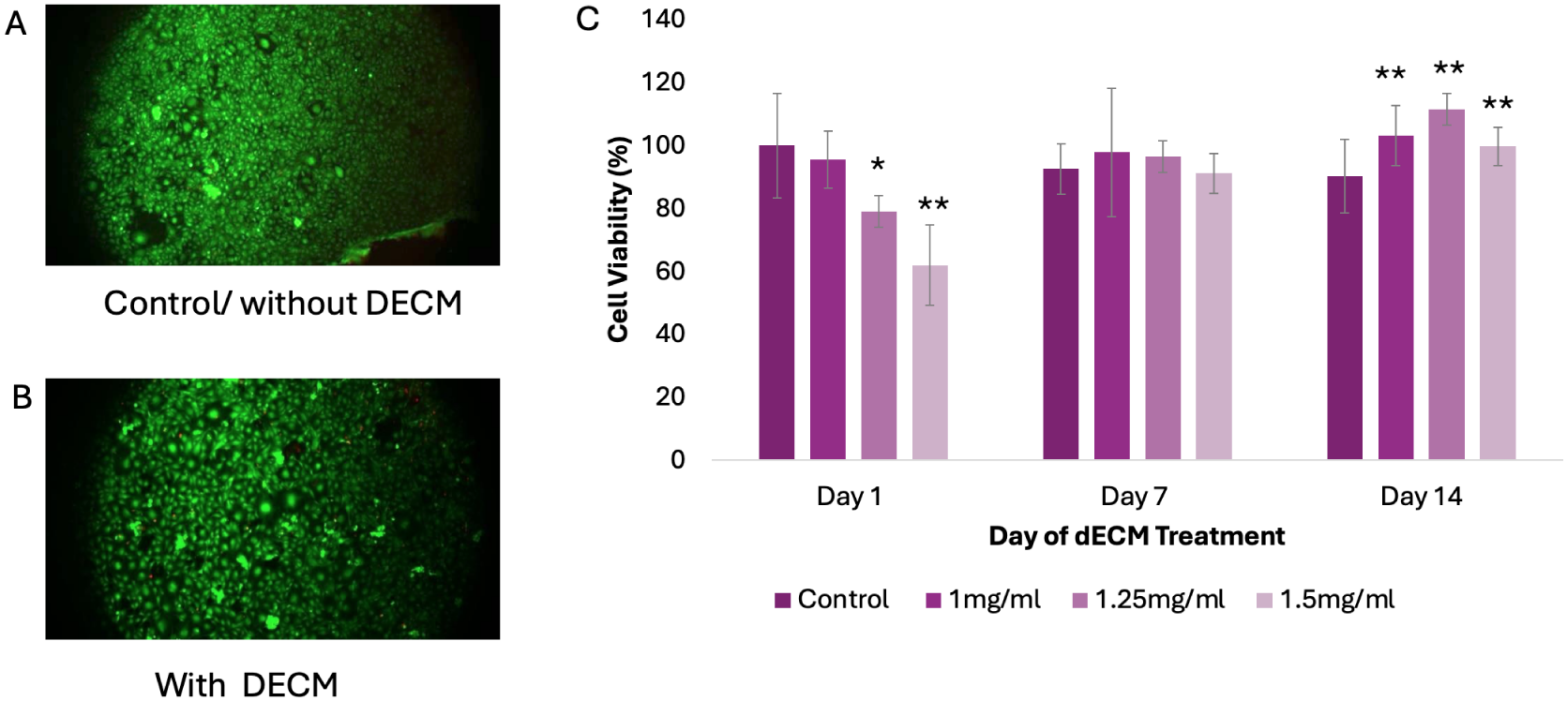
Effect of decellularized extracellular matrix (dECM) on metabolic activity of primary human osteoblasts (HOBs). (A, B) Representative live/dead fluorescence images of HOBs cultured for 14 days (A) without dECM (control) and (B) with dECM, showing predominantly viable cells (green) and a low proportion of nonviable cells (red). (C) Quantitative assessment of cellular metabolic activity using the Alamar Blue assay at days 1, 7, and 14 for HOBs cultured with dECM at concentrations of 1.0, 1.25, and 1.5 mg/mL, compared with untreated control. Data are presented as mean ± SD (*n* = 3). Statistical analysis was performed using two-way mixed ANOVA followed by one-way ANOVA with Bonferroni post hoc testing. A significant main effect of dECM concentration was observed (*F* (3,36) = 8.35, *p* < 0.001, partial η^2^ = 0.41). Alamar Blue measurements reflect cellular metabolic activity and do not directly distinguish between changes in cell number and metabolic state. *p < 0.05, **p < 0.01 versus control.

Subsequent one-way ANOVAs at each time point showed that on day 1, metabolic activity was significantly lower in the 1.25 mg/mL (*p* < 0.05) and 1.5 mg/mL (*p* < 0.01) groups compared with the control group. No significant differences were observed between groups on day 7 (*p* > 0.05). By day 14, metabolic activity was significantly higher in the 1.25 and 1.5 mg/mL groups compared with the control (*p* < 0.01).

### Alkaline Phosphatase (ALP) Expression and Activity

#### ALP Staining

Histochemical ALP staining was performed to assess the spatial distribution of ALP-positive regions in HOB cultures treated with different dECM concentrations (1.0, 1.25, and 1.5 mg/mL) and untreated controls. Representative images for days 7 and 14 are shown in Figure 7A–H. Quantification of the ALP-positive area indicated a significant main effect of dECM concentration at day 7 (*F*(3,25) = 4.41, *p* = 0.013, partial η^2^ = 0.35). Bonferroni post hoc tests identified significant differences between the 1.0 and 1.5 mg/mL groups and the control group (*p* < 0.05). At day 14, no significant differences were observed among treatment groups.

**Figure 7:**
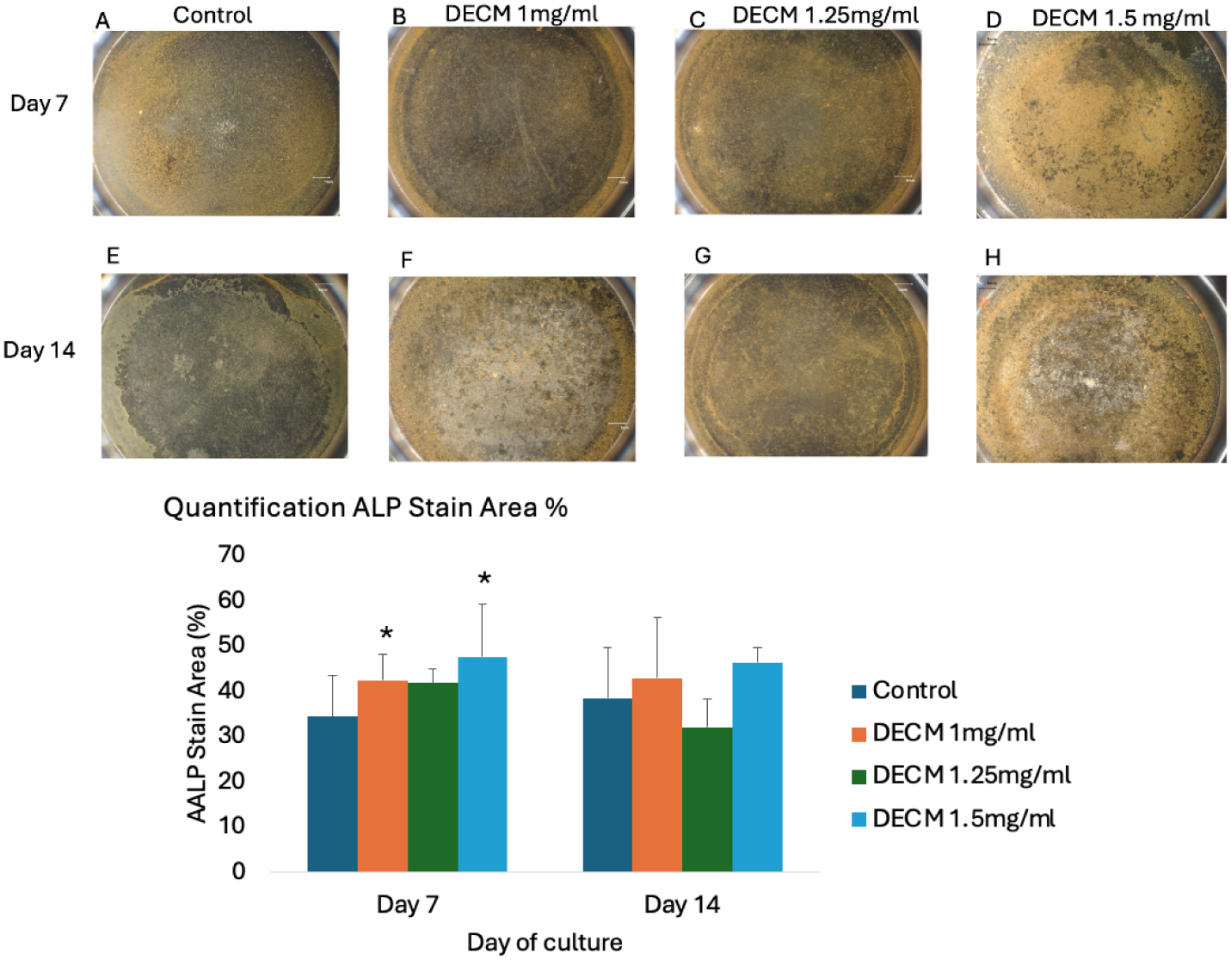
Alkaline phosphatase (ALP) staining of primary human osteoblasts (HOBs) cultured with decellularized extracellular matrix (dECM). (A–D) Representative ALP staining images at day 7 for HOBs cultured without dECM (control) and with dECM at concentrations of 1.0, 1.25, and 1.5 mg/mL. (E–H) Corresponding images at day 14. Red staining (Fast Red) indicates ALP-positive regions. Scale bar = 1 mm. Quantification of ALP-positive area at days 7 and 14. A significant increase in ALP-positive area was observed at day 7 in the 1.0 and 1.5 mg/mL groups compared with the control (*p < 0.05), whereas no significant differences were detected at day 14. Data are presented as mean ± SD.

#### Quantitative ALP Activity

ALP enzymatic activity was quantified using *p*-nitrophenyl phosphate substrate at 10- and 30-min reaction times (Figure 8).

**Figure 8:**
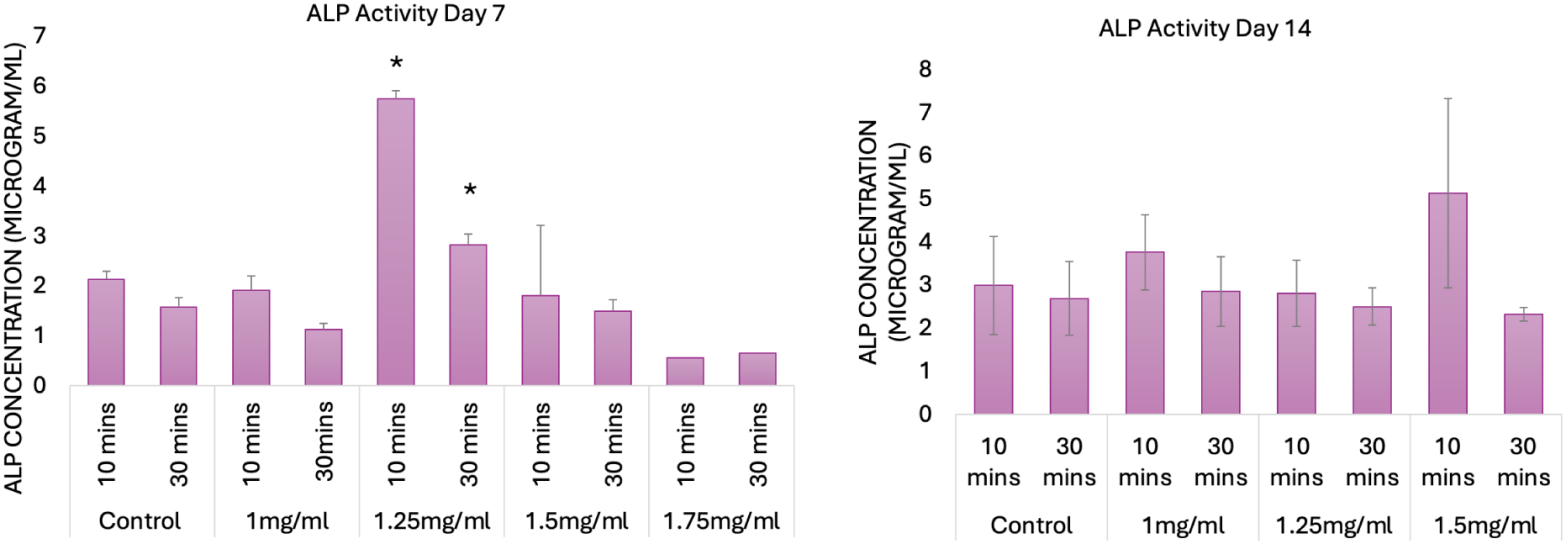
Quantitative assessment of alkaline phosphatase (ALP) activity in primary human osteoblasts (HOBs) cultured with different concentrations of decellularized extracellular matrix (dECM). ALP activity was measured using pNPP assay at day 7 (A) and day 14 (B) at 10 and 30 min. At day 7, a significant effect of dECM concentration was observed, with the 1.25 mg/mL group showing higher activity (*p < 0.05). No significant effect of concentration was detected at day 14. Data are presented as mean ± SD.

At day 7, Levene’s test indicated homogeneity of variance (*p* = 0.074). Two-way ANOVA showed a significant main effect of dECM concentration on ALP activity (*F*(3,52) = 4.93, *p* = 0.005, partial η^2^ = 0.236). Neither reaction time (*p* = 0.063) nor the interaction between concentration and reaction time (*p* = 0.294) reached significance. Post hoc Bonferroni analysis revealed that the 1.25 mg/mL group had significantly higher ALP activity than the control, 1.0 mg/mL, and 1.5 mg/mL groups (*p* < 0.05).

At day 14, Levene’s test indicated marginal variance homogeneity (*p* = 0.049). ANOVA showed no significant effect of dECM concentration on ALP activity (*p* = 0.208), while reaction time remained significant (*p* = 0.008). No significant interaction between concentration and reaction time was observed (*p* = 0.086).

### Expression of Osteogenic-Related Markers

RUNX2 gene expression at day 14 differed significantly among groups (one-way ANOVA, *F*(3,8) = 56.17, *p* < 0.001). Games–Howell post hoc analysis showed that the 1.25 mg/mL group exhibited significantly higher expression than all other groups. In contrast, the 1.0 mg/mL group showed moderate upregulation relative to the control, while the 1.5 mg/mL group showed reduced expression levels comparable to the control. ALP gene expression showed a similar trend, with increased expression at 1.25 mg/mL; however, the differences were less pronounced than those observed for RUNX2 (Figure 9).

**Figure 9:**
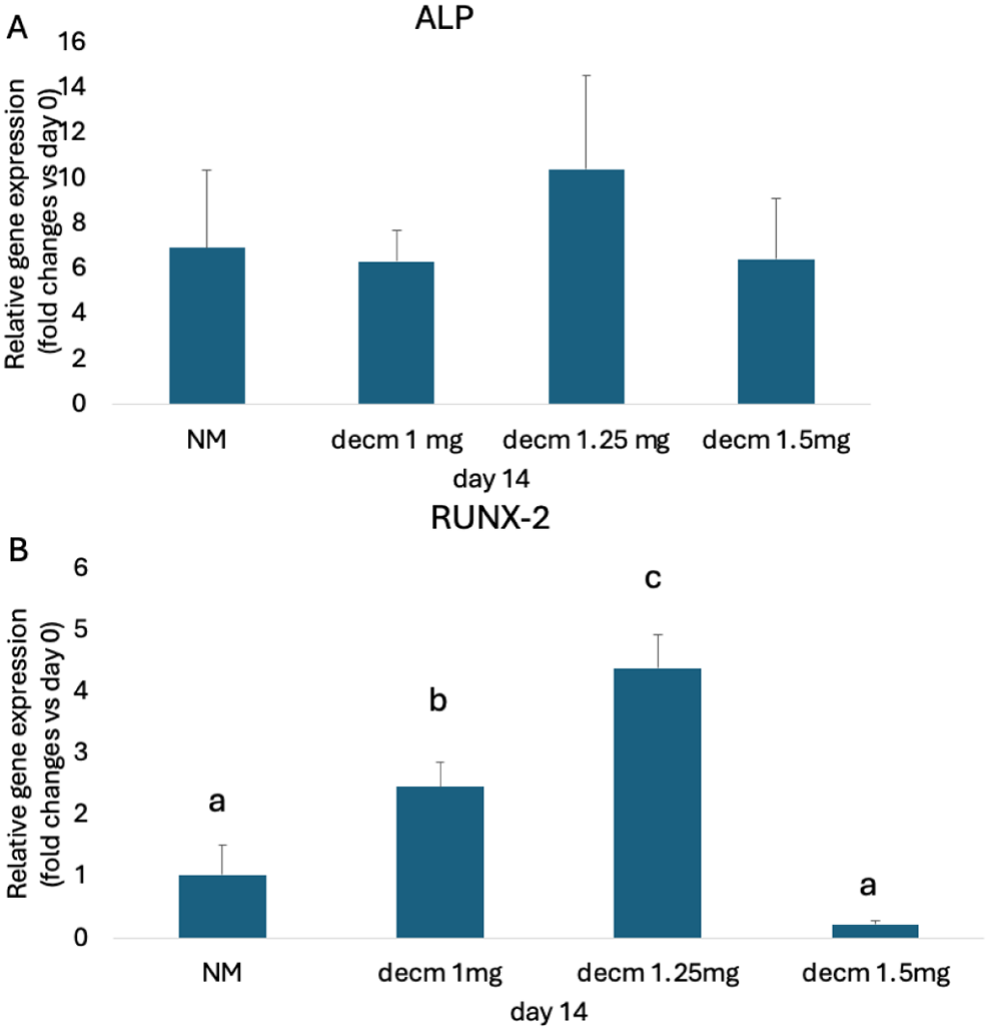
Effect of decellularized extracellular matrix (dECM) on osteogenic markers in primary human osteoblasts. (A) Relative alkaline phosphatase (ALP) gene expression at day 14. (B) Relative RUNX2 gene expression at day 14. Gene expression levels were quantified by qPCR and normalized to day 0. Cells were cultured with dECM at concentrations of 1.0, 1.25, and 1.5 mg/mL or without dECM (control/NM). Data are presented as mean ± SD (*n* = 3). Statistical analysis was performed using one-way ANOVA followed by Games–Howell post hoc test. Different letters indicate statistically significant differences between groups (*p* < 0.05).

Mineralization deposition was assessed on day 14 using von Kossa and Alizarin Red S staining to evaluate calcium and phosphate accumulation. Representative images are listed in Figure 10. Compared with control cultures, dECM-treated groups exhibited increased mineral deposition, as indicated by more intense and extensive staining in both assays. von Kossa staining revealed enhanced phosphate-rich nodules, while Alizarin Red S staining demonstrated increased calcium accumulation. These findings suggest that fibroblast-derived dECM supports progression toward matrix mineralization under the conditions tested.

**Figure 10:**
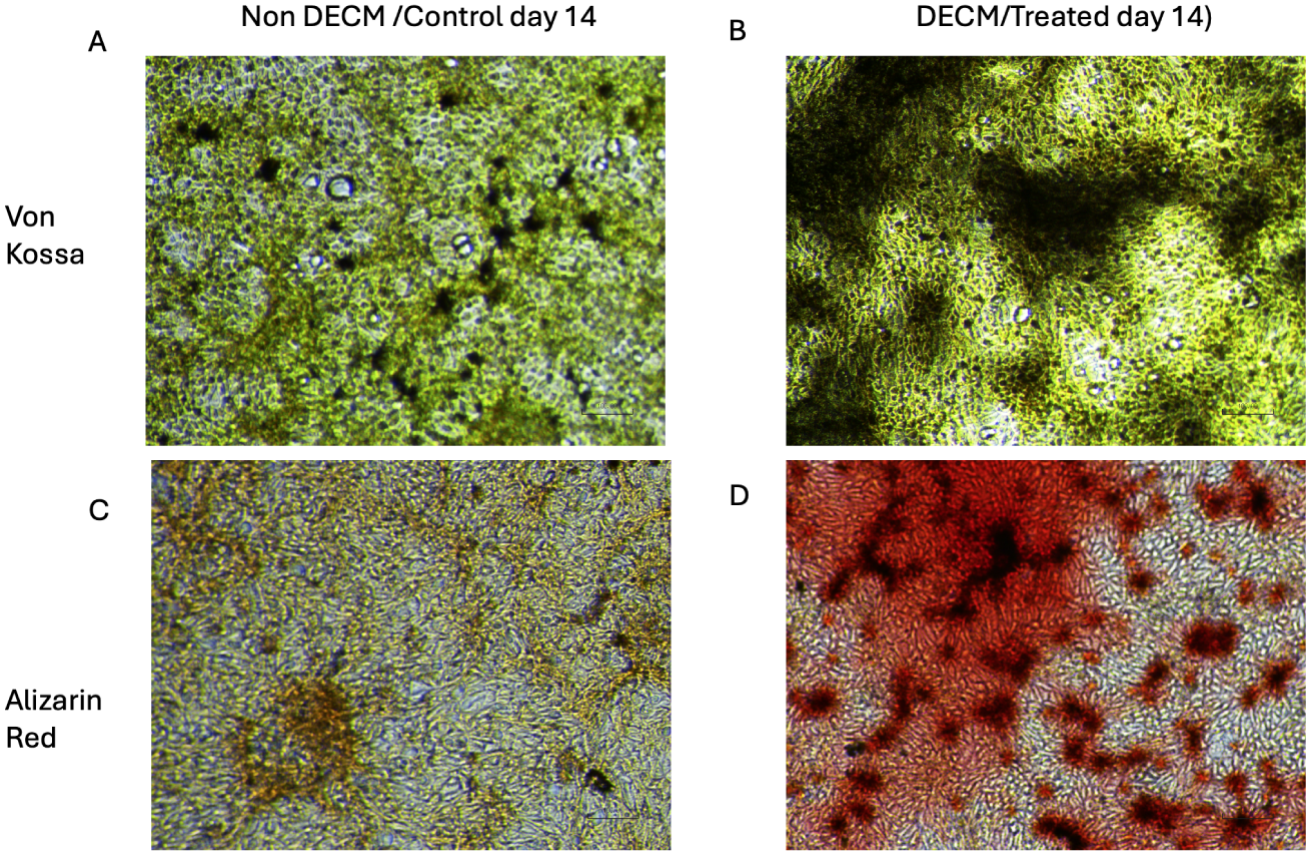
Mineralization of primary human osteoblasts cultured with and without decellularized extracellular matrix (dECM) at day 14. (A, B) von Kossa staining showing phosphate-rich mineral deposition in (A) control and (B) dECM-treated cultures. Dark/black regions indicate mineralized nodules. (C, D) Alizarin Red S staining showing calcium deposition in (C) control and (D) dECM-treated cultures. Red staining indicates calcium-rich deposits. Scale bar = 100 μm.

## Discussion

### Refinement of Decellularization Strategy

The present study establishes a refined detergent-based approach for generating fibroblast-derived decellularized extracellular matrix (dECM), achieving a substantial reduction in nuclear material while preserving key matrix components. Initial decellularization conditions resulted in incomplete nuclear removal and disruption of ECM architecture, consistent with previous reports indicating that insufficient detergent exposure fails to effectively lyse dense fibroblast cultures while compromising matrix integrity.^1,5^ Refinement of detergent concentration, exposure duration, and substrate stabilization improved DNA removal efficiency while maintaining protein content, highlighting the importance of balancing processing stringency with preservation of ECM structure.^7,8^

It is important to consider that refinement was performed across different culture durations, which likely influenced the ECM maturity and composition. Increased matrix deposition at later time points may contribute to enhanced structural stability and altered detergent accessibility. The refined protocol was further evaluated using 8-day cultures, which represent a more developed ECM environment compared to earlier time points. This approach enabled assessment of decellularization performance under conditions of increased matrix deposition. Under these conditions, lower residual DNA levels and reduced variability were observed. The 5-day condition was included to assess the effect of gelatin fixation within a more intermediate and reproducible ECM context. Increased matrix deposition at later time points may influence matrix structure and limit detergent access, which can affect decellularization efficiency. Therefore, improvements in decellularization efficiency should be interpreted in the context of both protocol refinement and differences in ECM maturity associated with culture duration.

DNA fragmentation analysis further demonstrated that residual DNA was predominantly below 100 bp, consistent with accepted decellularization criteria (<200 bp). Protein quantification further demonstrated that DNase treatment enhanced DNA removal but reduced total protein content, reflecting the known trade-off between nucleic acid clearance and ECM preservation.^14^ In contrast, detergent-based conditions without enzymatic supplementation showed improved protein retention, suggesting that controlled detergent exposure may provide a more balanced strategy for generating a biologically functional dECM.

### Proteomic Characterization and Matrix Composition

Proteomic analysis of osteoblast-derived ECM provided a contextual framework for identifying matrix-associated proteins relevant to osteogenic function. The detection of collagens, laminins, annexins, and CCN family proteins is consistent with established descriptions of the osteogenic matrisome and its role in regulating cell–matrix interactions.^2,3,12,14^

Calcium-binding proteins, including SPARC and annexins, were also identified and are known to contribute to mineral nucleation and early matrix maturation.^2,15,16^ Gene ontology enrichment further supported the functional relevance of these proteins, indicating associations with extracellular matrix organization, integrin binding, and calcium ion binding. Notably, histone and cytoskeletal proteins were detected in the decellularized matrix, suggesting the presence of residual intracellular components despite substantial DNA reduction. This observation highlights limitations in relying solely on bulk DNA quantification as a measure of decellularization efficiency and suggests that complementary approaches are required to fully assess matrix purity. It should also be noted that proteomic analysis was performed on pooled samples, limiting the interpretation to qualitative observations.

Furthermore, proteomics was conducted on an osteoblast-derived ECM rather than the fibroblast-derived ECM used in functional assays. Accordingly, these data should be interpreted as contextual rather than mechanistically definitive, providing a reference framework for matrix-associated components that are relevant to osteogenic function.

### Osteogenic Response of Human Osteoblasts to Decellularised ECM

Functional evaluation demonstrated that fibroblast-derived dECM modulates osteoblast behavior in a concentration-dependent and time-dependent manner. The intermediate concentration (1.25 mg/mL) produced the highest alkaline phosphatase (ALP) activity at day 7 and significantly increased RUNX2 expression at day 14, indicating enhanced early osteogenic signaling. ALP is widely recognized as an early marker of osteogenic differentiation, contributing to phosphate metabolism and the initiation of mineralization processes.^17^ However, ALP expression alone is insufficient to confirm progression to late-stage differentiation and may also reflect a transient metabolic adaptation. Therefore, the observed increase in ALP activity should be interpreted as indicative of early osteogenic signaling rather than definitive differentiation.

Differences observed between ALP enzymatic activity and histochemical staining likely reflect the distinct biological and methodological bases of the assays. While the *p*-nitrophenyl phosphate assay measures total enzymatic activity, histochemical staining detects spatially localized, membrane-associated ALP. These findings suggest that dECM concentration may influence both the magnitude and spatial distribution of ALP expression, consistent with previous reports that extracellular matrix composition and mechanical properties regulate osteogenic signaling and enzyme localization.^18^

In addition, mineralization assays (von Kossa and Alizarin Red S) performed on day 14 demonstrated increased calcium and phosphate deposition in dECM-treated cultures compared with controls. These findings support progression toward matrix mineralization under the conditions tested. Consistent with the observation, ECM derived from dental pulp stem cells has been shown to enhance mineralization in reseeded cells, even in the absence of osteogenic induction, highlighting the intrinsic bioactivity of cell-derived ECM in promoting mineral deposition.^2^ In the present study, although the fibroblast-derived ECM lacks a lineage-specific osteogenic composition, the observed increase in mineral deposition suggests that ECM structural and biochemical cues may contribute to osteogenic responses. However, these findings should be interpreted cautiously, as full osteogenic differentiation requires longer-term assessment and additional lineage-specific markers. Furthermore, the absence of comparative substrates or lineage-specific ECM controls limits the direct assessment of how these effects compare with commonly used substrates such as collagen or Matrigel.

Cell viability data further support a dynamic cellular response to dECM. The transient reduction in metabolic activity observed at early time points likely reflects initial cellular adaptation to the matrix environment, whereas increased metabolic activity at later stages suggests that dECM provides a supportive microenvironment for sustained cell function. Although a transient reduction in metabolic activity was observed at early time points, recovery at days 7 and 14 suggests that this effect reflects initial cellular adaptation rather than sustained cytotoxicity. This is consistent with previous reports describing dynamic cell–matrix interactions during early attachment and remodeling phases. Similar biphasic responses have been reported in ECM-based culture systems, where cells undergo an initial adjustment phase followed by enhanced proliferation and differentiation as cell–matrix interactions stabilize.^19–21^ In addition, fibroblast-derived dECM has been successfully incorporated into three-dimensional bioprinted hydrogel systems for osteochondral applications, where it supported enhanced cellular organization and tissue-specific responses,^22^ highlighting its versatility as a biomaterial platform beyond simplified in vitro models.

Overall, these findings demonstrate that fibroblast-derived dECM provides a biologically active substrate that modulates the early osteogenic responses. However, the current data primarily support regulation of early-stage signaling rather than definitive osteogenic differentiation, and further studies incorporating mineralization assays and advanced culture systems will be required to fully establish its functional potential.

### Limitations of the Study

Although this study establishes a refined decellularization protocol and demonstrates the biological activity of fibroblast-derived dECM in vitro, several limitations remain. First, all experiments were conducted in two-dimensional culture systems, which do not fully replicate the structural and mechanical complexities of the native bone microenvironment.

Second, osteogenic differentiation was assessed using both early markers (alkaline phosphatase activity and RUNX2 expression) and mineralization assays (Alizarin Red S and von Kossa staining). While increased calcium and phosphate deposition were observed in dECM-treated cultures, these findings represent progression toward matrix mineralization rather than definitive evidence of full osteogenic differentiation, which would require longer-term evaluation and additional lineage-specific markers.

Third, although substantial DNA reduction and fragmentation were achieved, with residual DNA predominantly below 100 bp, the presence of intracellular proteins detected by proteomic analysis suggests that the complete removal of cellular remnants was not achieved. This highlights the limitation of relying on DNA-based metrics alone to assess decellularization quality.

Finally, proteomic analysis was performed on pooled osteoblast-derived ECM samples and does not directly correspond to the fibroblast-derived ECM used in the functional assays. Therefore, these data are descriptive and contextual and do not establish mechanistic links to the observed cellular responses. Future studies incorporating quantitative proteomics of the fibroblast-derived ECM, three-dimensional culture systems, and in vivo validation will be important to further establish the translational potential of this approach.

## Conclusions

This study presents a refined detergent-based approach for the preparation of fibroblast-derived decellularized extracellular matrix (dECM), achieving substantial DNA removal while preserving key matrix components. Additional analysis demonstrated that residual DNA was highly fragmented, with sizes predominantly below 100 bp, consistent with established decellularization criteria.

Functional evaluation showed that dECM supports osteoblast viability and modulates osteogenic responses in a concentration-dependent manner, as evidenced by increased alkaline phosphatase activity and RUNX2 expression. Mineralization assays further demonstrated enhanced calcium and phosphate deposition in dECM-treated cultures, indicating progression toward matrix mineralization.

Overall, fibroblast-derived dECM provides a biologically active substrate capable of influencing osteoblast behavior. However, the findings remain limited to in vitro conditions, and further studies incorporating quantitative mineralization analysis, lineage-specific markers, and three-dimensional or in vivo models are required to fully establish its osteogenic potential and translational relevance.

## Materials and Methods

### Cell Culture and Extracellular Matrix (ECM) Preparation

Normal human dermal fibroblasts (NHDFs, adult) and primary human osteoblasts (HOBs) (PromoCell GmbH, C-12302 and C-12720) were cultured at 37 °C and 5% CO_2_. Fibroblasts were expanded in DMEM (D6546) supplemented with 10% fetal calf serum (FCS), 1% penicillin–streptomycin, and 1% l-glutamine. Osteoblasts were cultured in DMEM with phenol red (D6046) supplemented with 10% FCS, 10 mL HEPES, 1% l-glutamine, 2% penicillin–streptomycin, 5 mL MEM nonessential amino acids (M4526), and 0.075 g ascorbate powder.

Six-well plates were coated with 0.1% gelatin (Sigma-Aldrich, St. Louis, MO, USA), cross-linked with 0.1% glutaraldehyde, quenched with 0.1 M glycine, sterilized in 70% ethanol, washed, and equilibrated in culture medium before seeding. Fibroblasts were cultured for 5 or 8 days to allow ECM deposition.

### Decellularization Protocol

Decellularization of fibroblast-derived extracellular matrix (ECM) was performed using a modified detergent-based approach at defined culture time points to account for differences in matrix maturity. Preliminary experiments were conducted on day 4 to evaluate initial decellularization efficiency under conditions of limited ECM deposition. Subsequent optimization experiments were performed at later time points (days 5–8), allowing for increased matrix accumulation and improved structural integrity.

For decellularization, culture medium was removed, and samples were gently rinsed with phosphate-buffered saline (PBS) at room temperature (RT). Cells were treated with 1 mL of Triton X-100 solution (0.5–1% v/v; Sigma-Aldrich) for 30 min at 37 °C to disrupt cellular membranes. Triton X-100 was used at defined concentrations depending on the experimental condition (see Table 1), with 0.5% used in preliminary conditions and 1% used in refined protocols unless otherwise stated. This was followed by the addition of 1 mL ammonium hydroxide (NH_4_OH; 2 mM; Fisher Scientific) for 5 min at RT with gentle agitation to facilitate removal of nuclear material.

**Table 1:** Summary of Decellularization Conditions

Condition	Culture Day	Triton X-100	NH_4_OH	SDS	DNase	Duration	Substrate
Preliminary	Day 4	0.5%	1.4 mM (1/14 of 20 mM)	No	Tested (with and without DNase)	10 min	Noncross-linked
Tr	Day 5	0.5–1%	No	No	No	30 min	Cross-linked and noncross-linked
Am	Day 5	0.5–1%	2 mM	No	No	5 min	Cross-linked and noncross-linked
DNA	Day 5	0.5–1%	2 mM	No	Yes	45 min	Cross-linked and noncross-linked
SDS	Day 8	0.5–1%	2 mM	2%	No	10 min	Cross-linked and noncross-linked

**Table 2:** List of Primers

Gene	Manufacturer	Primer sequence
Beta-actin (ACTB)	Sigma-AldrichSY100817710–003/4	F-ATGAGGATGCTCACGGAGCGC GGCTACAGC
		R: ACACCACTGTGTTGGCGTACA GGTCTTTGC
Alkaline phosphatase (ALP)	Sigma-Aldrich NM_000478	F: AACACCACCCAGGGGAAC
		R: TGGCTGGTTCACTCTCGT
RUNX2	Sigma-Aldrich SY130325134–021	F-CCA ACC CAC GAA TGC ACT ATC
	Sigma-Aldrich SY130325134–022	R-TAG TGA GTG GTG GCG GAC ATA

In selected conditions, DNase I (50 μL mL^–1^, 45 min) was applied to further reduce residual DNA content. For additional matrix cleaning, sodium dodecyl sulfate (SDS; 2% w/v) was used for 10 min in later-stage experiments (day 8), where indicated.

Following treatment, all samples were carefully aspirated and washed 3–4 times with PBS at RT, with gentle agitation between washes to remove residual detergents and cellular debris. The resulting decellularized ECM (dECM) was either used immediately or stored in PBS at −80 °C prior to lyophilization.

Variations in detergent composition, exposure duration, and substrate stabilization were systematically adjusted across experimental groups to refine decellularization efficiency while preserving ECM structure. A summary of all conditions, including detergent concentrations, treatment durations, and substrate types, is provided in Table 1.

### dsDNA Quantification

Lyophilized dECM was scraped into sterile Bijou tubes, weighed, and resuspended in 500 μL PBS. DNA content was quantified using the Qubit High Sensitivity dsDNA Assay Kit (Invitrogen, Thermo Fisher Scientific) and measured on a Qubit 3.0 Fluorometer (Invitrogen). Ten microliters of sample were added to 190 μL of working solution, incubated for 2 min, and read according to the manufacturer’s instructions. DNA concentration (ng·mg^–1^) was calculated as

$$\mathrm{DNA}\left(\mathrm{ng}/\mathrm{mg}\right)=\frac{\mathrm{DNA concentration from Qubit}\left(\frac{\mathrm{ng}}{\mu l}\right)}{\mathrm{dry ECM mass}\left(\mathrm{mg}\right)}\times \mathrm{total extract volume}\left(\mu l\right)$$

For confirmatory analysis, total DNA was also measured on a NanoDrop 2000 spectrophotometer (Thermo Fisher Scientific, Waltham, MA, USA). Two microliters of the sample were analyzed at 260 nm using RNase-free water as the blank.

### PCR Amplification for DNA Fragment Detection

To further assess residual DNA, PCR amplification was performed targeting the ribosomal protein L19 (RPL-19) gene. Primer sequences were obtained from Sigma-Aldrich (SY190303046): forward primer 5′-CAG AAG ATA CCG TGA ATC T-3′ and reverse primer 5′-TGT TTT TTG AAC ACA TTC CCC-3′. PCR reactions were carried out using standard conditions with genomic DNA as the template. Amplified products were analyzed by agarose gel electrophoresis as described above to assess fragment size and the presence of residual DNA.

### DNA Fragmentation Analysis by Agarose Gel Electrophoresis

To assess DNA fragment size following decellularization, extracted DNA samples were analyzed by agarose gel electrophoresis. DNA was isolated from decellularized ECM using standard extraction procedures and quantified before analysis. Samples were mixed with loading dye and separated on a 2% (w/v) agarose gel prepared in tris-acetate-EDTA (TAE) buffer. A DNA ladder (100 bp) was used as a size reference. Electrophoresis was performed at 100 V for 30–40 min, and DNA bands were visualized using a gel imaging system following staining with a nucleic acid dye. Fragment size was estimated by comparison with the DNA ladder.

### Protein Quantification

Total protein was determined using the Pierce BCA Protein Assay Kit (Thermo Fisher Scientific, Waltham, MA, USA) according to the manufacturer’s instructions. Samples (50 μL) were mixed with 200 μL of working reagent, incubated at 37 °C for 30 min, and read at 560 nm using an Opsys MR microplate reader (Dynex Technologies, Chantilly, VA, USA).

### Effect of dECM on HOB Cells

To investigate the effect of decellularized extracellular matrix (dECM) on primary human osteoblast (HOB) cells, lyophilized dECM was reconstituted in osteoblast culture medium to prepare final concentrations of 1.0 mg/mL, 1.25 mg/mL, and 1.5 mg/mL. The resulting mixtures were added to HOB cultures and maintained for up to 14 days under standard culture conditions (37 °C, 5% CO_2_).

HOB cells (PromoCell GmbH, Heidelberg, Germany) were cultured in Dulbecco’s Modified Eagle Medium (DMEM; Sigma-Aldrich, D6046, Merck KGaA, Darmstadt, Germany) supplemented with 1 M HEPES buffer (Sigma-Aldrich, H0887, Merck KGaA, Darmstadt, Germany), 200 mM l-glutamine (Sigma-Aldrich, G7513, Merck KGaA, Darmstadt, Germany), 1% penicillin–streptomycin solution (Sigma-Aldrich, P0781, Merck KGaA, Darmstadt, Germany), 10% fetal calf serum (FCS; Sigma-Aldrich, F9665 Merck KGaA, Darmstadt, Germany), Minimum Essential Medium (MEM) nonessential amino acids (Sigma-Aldrich, M7145, Merck KGaA, Darmstadt, Germany), and ascorbate powder (Sigma-Aldrich, A0278, Merck KGaA, Darmstadt, Germany). The medium was changed every 2–3 days, and samples were collected at days 7 and 14 for further analyses, including viability, alkaline phosphatase activity, and gene expression. Each experimental condition (control, 1.0 mg/mL, 1.25 mg/mL, and 1.5 mg/mL dECM) was performed in triplicate, and the entire experiment was repeated independently twice to ensure reproducibility.

### Cell Viability Assay (Alamar Blue)

Metabolic activity was assessed using the Alamar Blue Cell Viability Reagent (Sigma-Aldrich, R7017, Merck KGaA, Darmstadt, Germany). The reagent was diluted 1:10 in phenol red-free Dulbecco’s Modified Eagle Medium (DMEM; Thermo Fisher Scientific, Waltham, MA, USA), sterile-filtered, and added to cultures (500 μL per well). Plates were incubated for 3 h at 37 °C and 5% CO_2_. Aliquots (200 μL) were transferred to 96-well plates, and absorbance was measured at 570 and 630 nm using a SpectraMax i3x plate reader (Molecular Devices, San Jose, CA, USA).

### Live/Dead Staining

Cell viability was confirmed using the LIVE/DEAD Viability/Cytotoxicity Kit (Thermo Fisher Scientific, Waltham, MA, USA). The working solution was prepared by adding 5 μL of calcein-AM and 20 μL of ethidium homodimer-1 to 10 mL of PBS. The medium was replaced with 300 μL of staining solution, and plates were incubated for 30 min at RT in the dark. Samples were imaged using an Olympus IX51 fluorescence microscope (Olympus Corporation, Tokyo, Japan).

### Alkaline Phosphatase (ALP) Activity

ALP activity was quantified using *p*-nitrophenyl phosphate (pNPP) substrate (Sigma-Aldrich, Merck KGaA, Darmstadt, Germany) on days 7 and 14. Fifty microliters of sample and 50 μL substrate were combined in 96-well plates, shaken for 2 min, and absorbance at 410 nm was recorded at 0, 10, and 30 min.

For histochemical analysis, cells were fixed in 4% paraformaldehyde (PFA; Sigma-Aldrich, Merck KGaA, Darmstadt, Germany) for 15 min, rinsed, and incubated with a staining solution containing naphthol AS-MX phosphate and Fast Red Violet LB (Sigma-Aldrich, Merck KGaA, Darmstadt, Germany) in Tris-HCl buffer for 45 min at RT. Stained plates were rinsed, air-dried and then imaged on a Keyence VHX-S750E digital microscope (Keyence Corporation, Osaka, Japan). The percentage-stained area was quantified by using Keyence analysis software under identical threshold parameters.

### Quantitative PCR (qPCR)

Total RNA was extracted using TRIzol reagent (Ambion, Thermo Fisher Scientific, Warrington, UK) and purified in Phase Lock Gel Heavy tubes (5 Prime, VWR International, Lutterworth, UK). RNA concentration and purity were assessed using a NanoDrop 1000 (Thermo Fisher Scientific, Waltham, MA, USA). Samples with A260/A280 ratios between 1.8 and 2.0 were used for cDNA synthesis. One microgram of RNA was reverse-transcribed using the QuantiTect Reverse Transcription Kit (Qiagen, West Sussex, UK).

Quantitative PCR (qPCR) was performed using a Stratagene MxPro real-time PCR system (Agilent Technologies, Santa Clara, CA, USA) with iTaq Universal SYBR Green Supermix (Bio-Rad Laboratories, Hercules, CA, USA). Amplification conditions were set according to the manufacturer’s protocol, and fluorescence data were collected at each cycle. The housekeeping gene Beta-Actin was used for normalization, and relative expression was calculated using the 2^–^ΔΔCt method. Primer sequences used for qPCR analysis are listed in Table 2.

### Immunofluorescence Staining

To assess ECM composition and decellularization efficacy, samples were fixed in 4% PFA for 15 min, permeabilised with 0.1% Triton X-100 (Sigma-Aldrich, Merck KGaA, Darmstadt, Germany) to allow antibody access to intracellular epitopes. This concentration is lower than that used during decellularization (0.5–1%) and was applied solely for permeabilisation. Samples were subsequently blocked with 3% fetal calf serum (Gibco, Thermo Fisher Scientific, Waltham, MA, USA) in PBS for 30 min. Fibronectin was detected using an antifibronectin primary antibody (Sigma-Aldrich, F3648 Merck KGaA, Darmstadt, Germany; 1:400, overnight at 4 °C) and an Alexa Fluor-conjugated antirabbit secondary antibody (Invitrogen, 6506111 Thermo Fisher Scientific, Waltham, MA, USA; 1:200, 1 h at RT). Nuclei were counterstained with Hoechst 33342 (Invitrogen, Thermo Fisher Scientific, Waltham, MA, USA, 1:1000, 10 min). Fluorescence images were acquired with an Olympus IX51 microscope using consistent exposure settings and analyzed in ImageJ (NIH, Bethesda, MD, USA).

### ALP Staining and Quantification

ALP histochemical staining was performed using naphthol AS-MX phosphate, and Fast Red Violet LB-stained wells were imaged using a Keyence VHX-S750E microscope with identical exposure settings across all samples.

Quantification of the ALP-positive area was carried out using Keyence analysis software. The “Measure” tool was selected, followed by the “Brightness (normal)” autoextraction mode. A circular region of interest (ROI) measuring 159 mm^2^ was manually drawn to match the inner diameter of each well and was used consistently for all images. No manual shape adjustments were applied. The software measured the ALP-positive area within the ROI, and the total ROI area was checked to confirm consistency across samples.

### Alizarin Red S Staining

Calcium deposition was assessed by using Alizarin Red S staining. On day 14, cultures were washed with phosphate-buffered saline (PBS) and fixed with 4% paraformaldehyde (PFA) for 15 min at room temperature (RT). Samples were rinsed with deionized water and incubated with 2% (w/v) Alizarin Red S solution (pH 4.2; Sigma-Aldrich) for 20 min at RT with gentle agitation. Excess dye was removed by repeated washing with deionized water until the background was clear. Stained samples were air-dried and imaged using a bright-field microscope under identical exposure conditions. Red staining indicates calcium-rich deposits.

### Von Kossa Staining

Phosphate deposition was evaluated by using von Kossa staining. On day 14, samples were washed with PBS and fixed with 4% paraformaldehyde (PFA) for 15 min at room temperature. The samples were then incubated with a 5% (w/v) silver nitrate solution and exposed to ultraviolet light for 30 min. After washing with deionized water, samples were treated with 5% sodium thiosulfate for 5 min to remove unreacted silver. Following final washes, samples were air-dried and imaged using bright-field microscopy. Black deposits indicate phosphate-rich mineralization.

### Protein Quantification and LC–MS /MS

Lyophilized treatment and control triplicates were pooled, reconstituted in 300 μL of deionized water, and quantified using a BCA assay. Samples were submitted to an accredited proteomics facility (Thermo Fisher Scientific Orbitrap Fusion Lumos, Waltham, MA, USA) for LC–MS/MS. Protein identifications and label-free quantification (LFQ) were performed using Proteome Discoverer 2.5 (Thermo Fisher Scientific, Waltham, MA, USA), and data were exported in .csv format for downstream analysis.

### Data Processing and Bioinformatics

Proteomic data were analyzed in R software (version 4.3.2; R Foundation for Statistical Computing, Vienna, Austria). Proteins were filtered at a false discovery rate (FDR) of *q* ≤ 0.01, with ≥2 unique peptides and ≥2 peptide-spectrum matches. LFQ intensities were log_2_-transformed, and fold changes were computed. Data visualization was conducted using the ggplot2 and pheatmap packages to produce violin plots and hierarchical heatmaps. Functional annotations were assigned via UniProt (https://www.uniprot.org) and Pfam.

### Gene Ontology and Pathway Analysis

Filtered protein data sets were cross-referenced against the Matrisome Project database (https://matrisomeproject.mit.edu) to extract ECM-associated proteins. These were submitted to the STRING v12.0 database (EMBL, Heidelberg, Germany) for gene ontology and protein–protein interaction analysis.

### Statistical Analysis

All quantitative data are presented as mean ± standard deviation (SD). Unless otherwise stated, n represents biological replicates. Statistical analyses were performed using SPSS software (IBM SPSS Statistics, version 31) and RStudio (RStudio Inc., Boston, MA, USA), as appropriate. A p-value <0.05 was considered statistically significant.

For comparisons involving more than two groups at a single time point, data were analyzed using one-way analysis of variance (ANOVA). Normality was assessed using the Shapiro–Wilk test, and homogeneity of variance was evaluated using Levene’s test. When equal variances were assumed, Tukey’s post hoc test was applied. When the assumption of homogeneity of variance was violated, Games–Howell post hoc testing was used.

For experiments involving the effects of both dECM concentration and time, two-way ANOVA was used, followed by Bonferroni-adjusted post hoc comparisons where appropriate. Where required, one-way ANOVA was subsequently performed at individual time points to assess the between-group differences.

For qPCR analysis, relative gene expression was calculated using the 2^−ΔΔCt method after normalization to the reference gene Beta Actin and calibration to day 0. Gene expression data were analyzed using one-way ANOVA, with appropriate post hoc testing assuming homogeneity of variances. Because of the small sample size, the results of assumption testing were interpreted cautiously.

Proteomic data were analyzed descriptively because biological replicates were pooled before LC–MS/MS analysis, precluding inferential statistical comparison between groups.

## Data Availability

The proteomics data generated in this study have been deposited in the PRIDE repository (https://www.ebi.ac.uk/pride/archive/projects/PXD077920) and are publicly available under accession number PXD077920

## References

[1] Guo Y., Zhang C.-Q., Zeng Q.-C., Li R.-X., Liu L., Hao Q.-X., Shi C.-H., Zhang X.-Z., Yan Y.-X. (2012). Mechanical
strain promotes osteoblast ECM formation and improves its osteoinductive
potential. BioMed. Eng. OnLine.

[2] Nowwarote N., Petit S., Ferre F. C., Dingli F., Laigle V., Loew D. (2022). Extracellular matrix derived from dental pulp stem
cells promotes mineralization. Front. Bioeng.
Biotechnol..

[3] Jeon J., Lee M. S., Yang H. S. (2018). Differentiated
osteoblasts derived
decellularized extracellular matrix to promote osteogenic differentiation. Biomater. Res..

[4] Kim H.-S., Hwang H.-J., Kim H.-J., Choi Y., Lee D., Jung H.-H. (2022). Effect
of decellularized extracellular matrix bioscaffolds
derived from fibroblasts on skin wound healing and remodeling. Front. Bioeng. Biotechnol..

[5] Vilaça-Faria H., Noro J., Reis R. L., Pirraco R. P. (2024). Extracellular
matrix-derived
materials for tissue engineering and regenerative medicine: A journey
from isolation to characterization and application. Bioactive Mater..

[6] Vavken P., Joshi S., Murray M. M. (2009). TRITON-X
is most effective among
three decellularization agents for ACL tissue engineering. J. Orthop. Res..

[7] Harris, G. M. ; Raitman, I. ; Schwarzbauer, J. E. Cell-derived decellularized extracellular matrices. In Methods In Cell Biology; Elsevier: 2018,Vol: 143, pp. 97–114. DOI: 10.1016/bs.mcb.2017.08.007. 29310794 PMC5995326

[8] Neishabouri A., Soltani Khaboushan A., Daghigh F., Kajbafzadeh A.-M., Majidi Zolbin M. (2022). Decellularization in tissue engineering and regenerative
medicine: evaluation, modification, and application methods. Front. Bioeng. Biotechnol..

[9] Golebiowska A. A., Intravaia J. T., Sathe V. M., Kumbar S. G., Nukavarapu S. P. (2024). Decellularized
extracellular matrix biomaterials for regenerative therapies: advances,
challenges and clinical prospects. Bioactive
Mater..

[10] Hanai H., Jacob G., Nakagawa S., Tuan R. S., Nakamura N., Shimomura K. (2020). Potential of soluble decellularized extracellular matrix
for musculoskeletal tissue engineering–comparison of various
mesenchymal tissues. Front. Cell Dev. Biol..

[11] Tracy L. E., Minasian R. A., Caterson E. (2016). Extracellular
matrix and dermal fibroblast
function in the healing wound. Adv. Wound Care.

[12] Naba A. (2023). Ten years
of extracellular matrix proteomics: Accomplishments, challenges, and
future perspectives. Mol. Cell. Proteomics.

[13] Katsavelis D., van der Hart M. G. C., Wolters J. C., Permentier H. P, Horvatovich P., Cremers T. I. F. H. (2025). Impact of Sample Preparation Strategies
on the Quantitative Accuracy of Low-Abundance Serum Proteins in Shotgun
Proteomics. J. Proteome Res..

[14] Monteiro-Lobato G. M., Russo P. S. T., Winck F. V., Catalani L. H. (2022). Proteomic analysis
of decellularized extracellular matrix: achieving a competent biomaterial
for osteogenesis. BioMed. Res. Int..

[15] Mortada I., Mortada R. (2018). Dental pulp stem cells and osteogenesis:
an update. Cytotechnology.

[16] Bradshaw A. D. (2009). The role
of SPARC in extracellular matrix assembly. J.
Cell Commun. Signaling.

[17] Vimalraj S. (2020). Alkaline phosphatase:
Structure, expression and its function in bone mineralization. Gene.

[18] Na J., Yang Z., Shi Q., Li C., Liu Y., Song Y. (2024). Extracellular matrix
stiffness as an energy metabolism
regulator drives osteogenic differentiation in mesenchymal stem cells. Bioactive Mater..

[19] Guo Y., Zeng Q., Yan Y., Shen L., Liu L., Li R., Zhang X., Wu J., Guan J., Huang S. (2013). Proliferative
effect and osteoinductive potential of extracellular matrix coated
on cell culture plates. Springerplus.

[20] Blair H. C., Larrouture Q. C., Li Y., Lin H., Beer-Stoltz D., Liu L. (2017). Osteoblast differentiation
and bone matrix formation
in vivo and in vitro. Tissue Eng. Part B.

[21] Lin X., Patil S., Gao Y.-G., Qian A. (2020). The bone extracellular
matrix in bone formation and regeneration. Front.
Pharmacol..

[22] Garna D. F., Shet A. S., Randall Morgan L. R., Di-Silvio L. (2026). Fibroblast
Matrix Enhanced Three-Dimensional-Bioprinted Hydrogel for Osteochondral
Regeneration. Tissue Eng. Part A.

